# Association between K469E polymorphism of ICAM‐1 gene and susceptibility of ischemic stroke: An updated meta‐analysis

**DOI:** 10.1002/mgg3.784

**Published:** 2019-06-03

**Authors:** Gaurav Nepal, Jayant Kumar Yadav, YuHui Kong

**Affiliations:** ^1^ Tribhuvan University Institute of Medicine Kathmandu Nepal; ^2^ Chengdu University of Information Technology Chengdu Sichuan China

**Keywords:** cerebral infarct, ICAM‐1; K469E, ischemic stroke, Lys469Glu; rs5498, polymorphism

## Abstract

**Background:**

The intercellular adhesion molecule‐1 (ICAM‐1)/leukocyte function associated antigen‐1 (LFA‐1) adhesion system regulates leukocyte interactions, migration, and adhesion, and appears to play an important role in atherosclerosis and thrombosis. Therefore, single nucleotide polymorphisms (SNPs) of the ICAM‐1 gene may strongly influence the expression and biological activity of ICAM‐1 and play a potentially important role in the pathogenesis of ischemic stroke. In the current meta‐analysis, we investigated the relationship between the ICAM‐1 gene K469E SNP and the risk of ischemic stroke.

**Methods:**

Two investigators independently searched PubMed, Web of Science, Google Scholar, WANFANG, China National Knowledge Infrastructure (CNKI) and J‐STAGE for studies published from January 2000 to February 2019 without language restriction. The association of K469E polymorphism and ischemic stroke in three genetic models (allelic, recessive, and dominant) were evaluated using Pooled odds ratios (ORs) with 95% confidence intervals (CIs).

**Results:**

Our study included 20 studies from four continents and four different countries, including 3,137 cases and 15,382 controls. Meta‐analysis results did not show a significant association between K469E polymorphism of ICAM‐1 gene and ischemic stroke when assuming allelic model (OR: 1.12; 95% CI: 0.8 to 1.55; *p* = 0.51; *I* ^2^ = 93%) or recessive model (OR: 1.28; 95% CI: 0.89 to 1.84; *p* = 0.18; *I* ^2^ = 82%) or dominant model (OR: 1.20; 95% CI: 0.92 to 1.56; *p* = 0.17; *I* ^2^ = 85%). However, in all three genetic models, subgroup analysis revealed that the K469E polymorphism of the ICAM‐1 gene is associated with ischemic stroke in the Caucasian population.

**Conclusion:**

K469E polymorphism of ICAM‐1 gene might be a risk factor for ischemic stroke in Caucasians, which suggested that K469E polymorphism might help in early identification of those at risk and help in primary prevention of ischemic stroke.

## 1 BACKGROUND

Globally, stroke is the second leading cause of death after ischemic heart disease and the leading cause of long‐term disability. Approximately, 80% of new strokes are ischemic in origin, 51% stroke death, and 58% of stroke Disability‐Adjusted Life Year (DALY) are because of ischemic stroke (Feigin et al., 2015). Ischemic stroke is thought to be the result of interactions between genetic and environmental factors (Boehme, Esenwa, & Elkind, 2017). There is strong evidence from studies that genetic susceptibility, in addition to recognized risk factors such as hypertension, smoking, diabetes, obesity, and advanced age, contributes to the development of stroke (Boehme et al., 2017). In addition, people with a family history of stroke are more likely to have a stroke. Therefore, understanding these genetic influences may lead to better prevention and intervention of stroke.

Vascular inflammation, characterized by the recruitment and adhesion of circulating leukocytes by cell adhesion molecules in the endothelium, plays an important role in the pathogenesis of atherosclerosis (Galkina & Ley, 2007). Intercellular adhesion molecule‐1 (ICAM‐1), a member of the immunoglobulin superfamily, is a surface glycoprotein expressed on the vascular endothelium and is the major ligand for leukocyte function‐associated antigen‐1 (LFA‐1), a member of the integrin superfamily (Wee, Oh, Jo, & Jun, 2009). The ICAM‐1/LFA‐1 adhesion system regulates leukocyte interactions, migration and adhesion and appears to play an important role in early atherosclerosis (Wee et al., 2009). Therefore, ICAM‐1 is an important factor in thrombosis and may play an important role in ischemic stroke. The human ICAM1 gene is located on chromosome 19p13.3 to 13.2, spanning 15.5 kb of genomic DNA, including 7 exons and 6 introns (Vora, Rosenbloom, Beaudet, & Cottingham, 1994). K469E/Lys469glu/rs5498 is a single nucleotide polymorphism (SNP) located in exon 6 of the ICAM‐1 gene (Vora et al., 1994). It occurs due to the C to T polymorphism, resulting in a change in glutamate (E) to lysine (K) in the immunoglobulin‐like domain 5 of the ICAM‐1 protein (Vora et al., 1994). The SNP of ICAM‐1 gene may strongly influence the expression and biological activity of ICAM‐1, and has a potentially important role in the pathogenesis of ischemic stroke.

Multitudes of studies have been conducted to determine the association between K469E SNPs and ischemic stroke, but their results are inconsistent. A meta‐analyses of 12 studies have been conducted previously to elucidate the association between K469E polymorphism and the risk of ischemic stroke (Zhang et al., 2014). However, the meta‐analysis included only Chinese studies in their analysis. To the best of our knowledge, this is the largest meta‐analysis, including Caucasian, Black, and Asian population, conducted till now to determine the association between K469E polymorphisms and risk of ischemic stroke. Our meta‐analysis differs from previous meta‐analysis by inclusion of 20 studies, inclusion of major ethnicity, inclusion of more recent studies. Therefore, we chose to update the meta‐analysis of this issue to provide more accurate evidence. In this study, we conducted extensive review of the literature and conducted a meta‐analysis to assess the relationship between the ICAM‐1 gene K469E polymorphism and susceptibility to ischemic stroke.

## 2 METHODS

### 2.1 Ethical compliance

All collected data were extracted from published studies, and there is no ethical issue. This current meta‐analysis was conducted in accordance with the guidance of the Preferred Reporting Items for Systematic Reviews and Meta‐Analyses (PRISMA) statement (Liberati et al., 2009).

### 2.2 Literature search

We searched PubMed, Web of Science, Google Scholar, WANFANG, China National Knowledge Infrastructure (CNKI), and J‐STAGE for studies published January 2000–February 2019. Searches were conducted using the keywords ‘“ICAM‐1 gene polymorphism”’ or “K469E polymorphism “or “rs5498 polymorphism” or ‘“Lys469glu polymorphism”’ in combination with “stroke” or ‘“ischemic stroke”’ or ‘“cerebrovascular disease”’ or ‘“cerebral infarction”’. Titles and abstracts were then screened and the full text of all the papers that were considered to possibly meet the inclusion criteria was obtained. The cited references of retrieved articles were manually checked to identify any additional eligible studies. Two authors (GN and YK) screened and retrieved reports and excluded irrelevant studies. Any uncertainty about the eligibility of these studies were solved by discussion with all the authors.

### 2.3 Eligibility criteria

We included studies that were conducted on human subjects and the studies were searched without any limitations on language. In this meta‐analysis, all included studies met the following criteria: (a) case–control studies focused on the association between K469E polymorphism and ischemic stroke susceptibility; (b) patients with ischemic stroke were diagnosed with neuroimaging confirmed by neurologist; and (c) there were sufficient data of the genotypes in the case–control groups to evaluate the ORs and 95% CIs. The exclusion criteria were: (a) publications with overlapping cases and controls from the similar study; (b) no genotypic data available; and (c) publications with patients with cardioembolic ischemic stroke or hemorrhagic stroke.

### 2.4 Data abstraction and assessment of methodological quality

The relevant data from each study were independently extracted by two reviewers (GN and YK) using a standardized, structured form including the first author, type of design, site of study, patient ethnicity, year of publication, sample size of cases and controls, control design, Hardy–Weinberg equilibrium, source of DNA, genotyping method, and data of the genotypes in the case–control groups. The methodological quality of each study was assessed independently by two reviewers (GN and YK) using the parameters previously used by (Kumar et al., 2016). In this scale, following sections were included for assessment of methodological quality: representativeness of cases, source of controls, matching of controls, ascertainment of cases, ascertainment of controls, blinding while genotyping, genotyping methods, Hardy–Weinberg equilibrium, and association assessment. Maximum score of 2 can be provided in each sections, except source of controls (maximum 3), ascertainment of controls (maximum 1), blinding while genotyping (maximum1), and association assessment (maximum1). Methodological quality of scale score varies from 0 to 16, in which higher score represents the better quality and lower score represents the lower quality. Any discrepancies during data extraction and quality assessment were resolved by discussion with all the authors.

### 2.5 Statistical analysis

The association between K469E polymorphism and ischemic stroke susceptibility was evaluated by calculating the pooled ORs and 95% CIs. Heterogeneity between the included studies was first determined with Cochran's *Q* test and *I* ^2^ test. The presence of *P* values above 0.1 or *I* ^2^ more than 50%, was considered as an indicator of significant heterogeneity. When significant heterogeneity was present, we selected the random‐effects model to calculate the effects size. Three genetic models were examined, the allelic model (E allele vs. K allele), the dominant model (EE + EK vs. KK), and recessive model (EE vs. EK + KK). Subgroup analyses were conducted according to ethnicity and methodological quality evaluation. Sensitivity analysis was performed to examine the stability of analysis. Meta‐regression was performed to determine whether methodological quality of study have role in effect size associated with K469E polymorphism. Egger's linear regression test and Begg's test were used for the identification of publication biases. Meta‐analysis, Meta‐regression, Egger's linear regression test, and Begg's test were performed in Comprehensive Meta‐Analysis software (CMA 3.3, Biostat, Englewood, NJ, 2014).

## 3 RESULTS

### 3.1 Literature search

**Figure 1:**
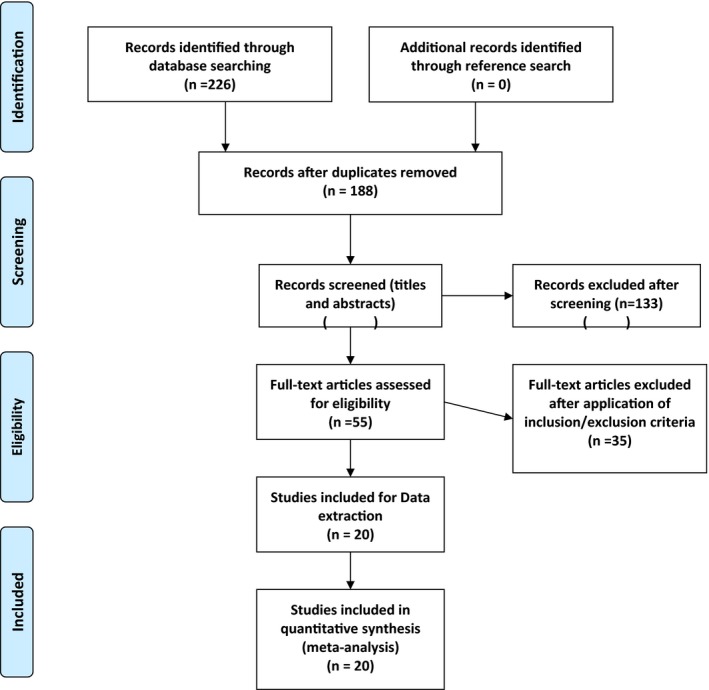
Flow of systematic literature search and selection

The results of the systematic literature search and selection are summarized in Figure 1. We identified 226 articles through database searching. After exclusion of duplicates, 188 articles remained and after screening titles and abstracts, 55 relevant abstracts remained. After application of inclusion and exclusion criteria, 20 studies were chosen for final analysis and the data were extracted.

### 3.2 Study and patient characteristics

Our research included 20 studies from four continents and four different countries and included 3,137 cases and 15,382 controls. Fifteen studies were from China (Geng, 2011; Guo, Liu, & Guo, 2011; Huihua et al., 2016; Li et al., 2009; Liu, 2005; Shang, 2004; Sun & Zhang, 2005; Sun, Wu, & Deng, 2009; Wang et al., 2015; Wei, Liu, Huang, Tang, & Meng, 2005; Xing, Song, Hongjun, & Zhang, 2006; You, Huang, & Liu, 2007; Zhang, Cheng, Peng, Wang, & Peng, 2007; Zhang & Zhang, 2012; Zhou, Zhu, & Sun, 2007), two studies were from Italy (Flex et al., 2004; Pola et al., 2003), two studies from Egypt (Mohy, Ali, & Omar, 2015; Motawi, Shaker, Taha, & Abdel Raheem, 2012), one study was from the United States (Volcik, Ballantyne, Hoogeveen, Folsom, & Boerwinkle, 2010). Caucasian subjects were included in six studies (Flex et al., 2004; Geng, 2011; Mohy et al., 2015; Motawi et al., 2012; Pola et al., 2003; Volcik et al., 2010), black subjects were included in one (Volcik et al., 2010), and Asians were included in rest of the studies. The publication date ranged from 2003 (Pola et al., 2003) to 2016 (Huihua et al., 2016). All included studies have case–control designs. In all of the included studies, Hardy–Weinberg equilibrium was reached in the control population except in one (Zhou et al., 2007). Polymerase chain reaction‐restriction fragment length polymorphism (PCR‐RFLP) was most common form of genotyping. However, some studies used TaqMan PCR (Volcik et al., 2010), T‐gradient PCR (Motawi et al., 2012), and TaKaRa PCR kits (Wang et al., 2015). Controls were population‐based in the 16 studies but hospital based in four (Flex et al., 2004; Liu, 2005; Pola et al., 2003; Zhang et al., 2007). Allelic data were not available in one study (Volcik et al., 2010) and genotypic data were incomplete in one study (Mohy et al., 2015). According to quality assessment tools, the methodological quality score ranged from 8 to 12. None of the study were of low quality. All study and patient characteristics are tabulated in Table 1.

**Table 1:** Key methodological characteristics of studies included in this meta‐analysis

Author	Year	Country	Ethnicity	Source of controls	Sample size (case/control)	Genotyping method	Genotypes distribution (case/control)			Allelic distribution (case/control)		HWE	Quality score
							KK	EK	EE	E	K		
Pola	2003	Italy	Caucasian	HB	119/133	PCR‐RFLP	24/49	63/68	32/16	127/100	111/166	Yes	12
Shang	2004	China	Asian	PB	53/71	PCR‐RFLP	25/42	17/24	11/5	39/34	67/108	Yes	11
Flex	2004	Italy	Caucasian	HB	237/223	PCR‐RFLP	53/75	112/125	72/23	256/171	218/275	Yes	12
Sun	2005	China	Asian	PB	76/105	PCR‐RFLP	42/42	30/44	4/19	38/82	114/128	Yes	11
Wei	2005	China	Asian	PB	205/210	PCR‐RFLP	74/95	99/97	32/18	163/133	247/287	Yes	11
Liu	2005	China	Asian	HB	142/101	PCR‐RFLP	65/50	65/44	12/7	89/58	195/144	Yes	9
Xing	2006	China	Asian	PB	112/105	PCR‐RFLP	59/29	35/44	18/32	71/108	153/102	Yes	10
You	2007	China	Asian	PB	177/112	PCR‐RFLP	72/62	86/40	19/10	124/60	230/164	Yes	9
Zhou	2007	China	Asian	PB	92/121	PCR‐RFLP	37/61	39/41	16/19	71/79	113/163	No	9
Zhang	2007	China	Asian	HB	309/309	PCR‐RFLP	149/192	131/102	29/15	189/130	429/488	Yes	11
Sun	2009	China	Asian	PB	92/110	PCR‐RFLP	48/31	29/46	15/33	58/114	126/106	Yes	11
Li	2009	China	Asian	PB	309/309	PCR‐RFLP	148/192	132/102	29/15	190/132	428/486	Yes	12
Volcik	2010	USA	Caucasian	PB	290/9593	TaqMan PCR	99/3111	138/4836	53/1646	N/A	N/A	Yes	11
Volcik	2010	USA	Black	PB	223/3181	TaqMan PCR	159/2129	60/942	4/110	N/A	N/A	Yes	11
Geng	2011	China	Caucasian	PB	100/110	PCR‐RFLP	65/52	23/43	12/15	47/73	153/147	Yes	10
Guo	2011	China	Asian	PB	115/99	PCR‐RFLP	35/50	60/42	20/7	100/56	130/142	Yes	9
Zhang	2012	China	Asian	PB	120/102	PCR‐RFLP	28/39	52/46	40/17	132/80	108/124	Yes	12
Motawi	2012	Egypt	Caucasian	PB	63/75	T‐ gradient PCR	21/45	15/21	27/9	69/39	57/111	Yes	11
Mohy	2015	Egypt	Caucasian	PB	40/40	PCR‐RFLP	7/25	N/A	N/A	35/15	45/65	Yes	8
Wang	2015	China	Asian	PB	50/50	TaKaRa PCR kits	41/35	4/7	5/8	14/27	86/73	No	10
Jiang	2016	China	Asian	PB	213/223	PCR‐RFLP	111/76	86/88	26/60	128/207	170/34	Yes	12

Abbreviations: PB: Population based; HB: Hospital based; N/A: Not available; HWE: Hardy–Weinberg equilibrium; PCR‐RFLP: Polymerase chain reaction‐restriction fragment length polymorphism

### 3.3 Meta‐analysis

**Figure 2:**
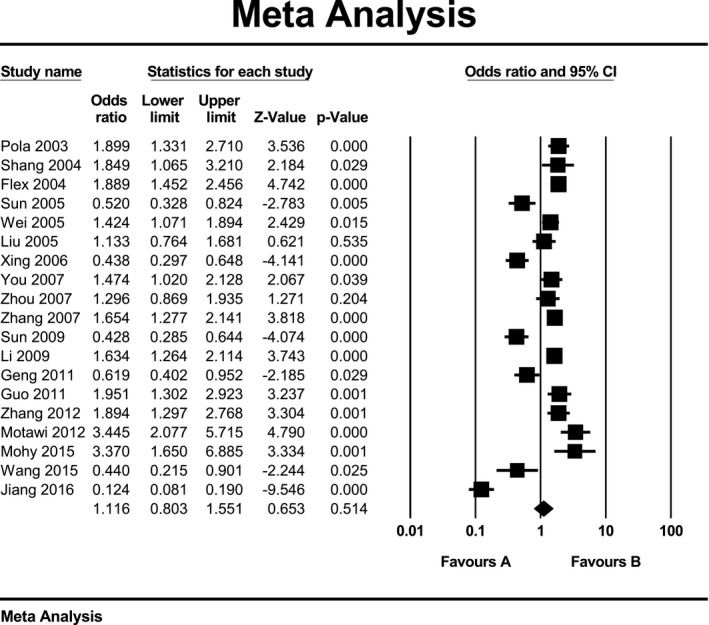
Forest plot of the result for allelic model

**Figure 3:**
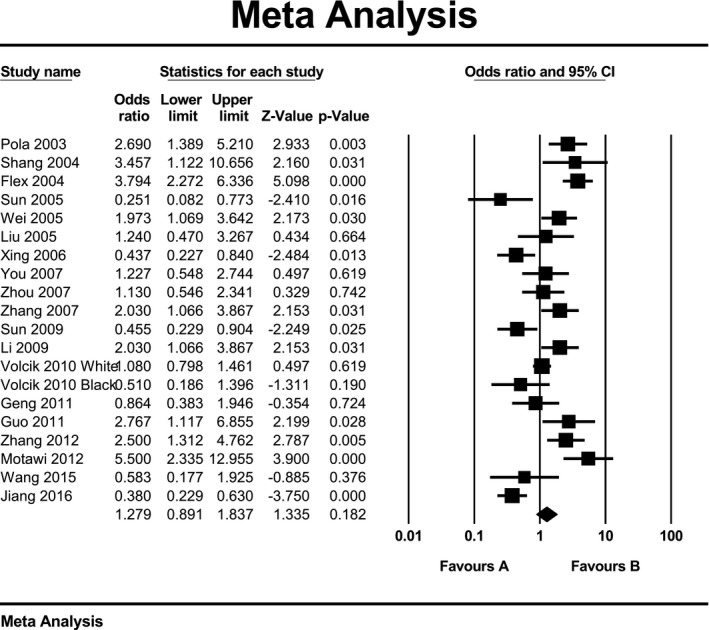
Forest plot of the result for recessive model

**Figure 4:**
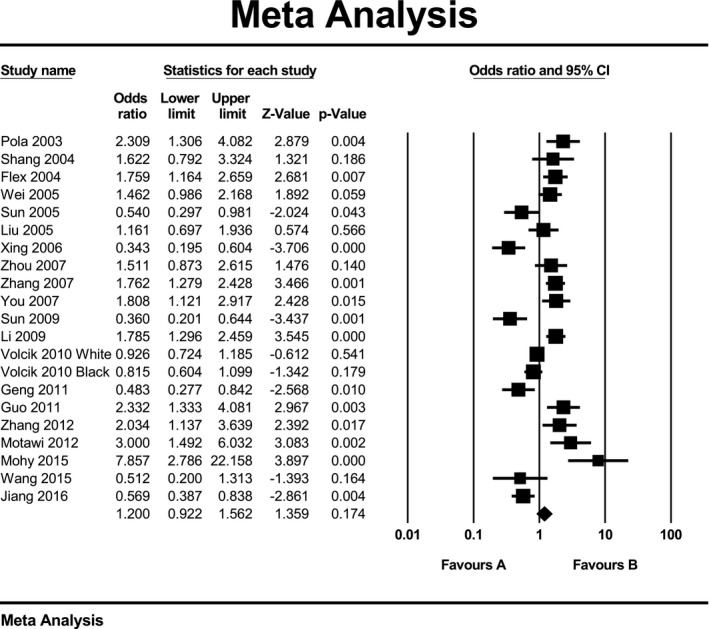
Forest plot of the result for dominant model

**Figure 5:**
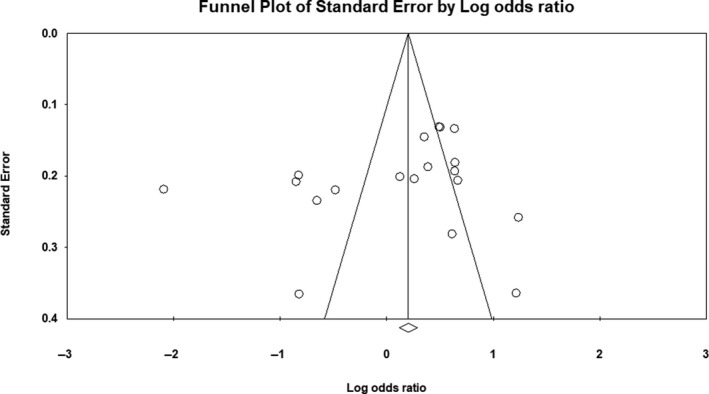
Funnel plot for detection of publication bias in allelic model

**Figure 6:**
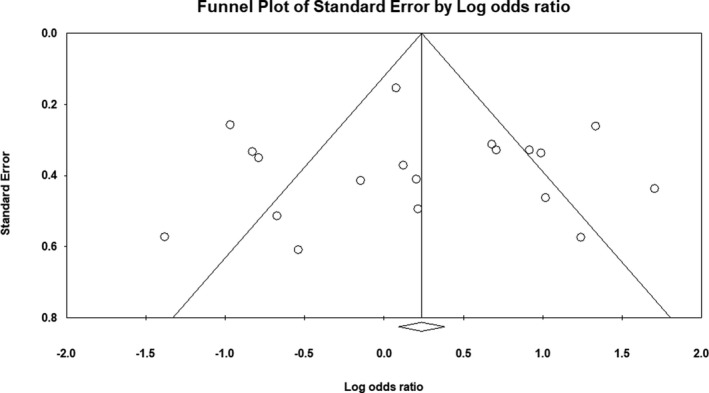
Funnel plot for detection of publication bias in recessive model

**Figure 7:**
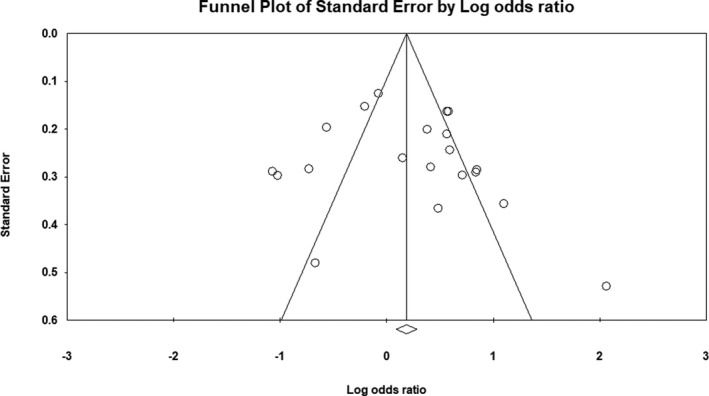
Funnel plot for detection of publication bias in dominant model

Due to high heterogeneity within studies in all three genetic models, we used random‑effects model to estimate pooled ORs and 95% CIs. Meta‐analysis results did not show a significant association between K469E polymorphism of ICAM‐1 gene and ischemic stroke when assuming allelic model (OR: 1.12; 95% CI: 0.8 to 1.55; *p* = 0.51; *I* ^2^ = 93%) or recessive model (OR: 1.28; 95% CI: 0.89 to 1.84; *p* = 0.18; *I* ^2^ = 82%) or dominant model (OR: 1.20; 95% CI: 0.92 to 1.56; *p* = 0.17; *I* ^2^ = 85%). Forest plot of the result for allelic, recessive, and dominant model is demonstrated in Figures 2, 3, 4 respectively. There was no evidence of publication bias on allelic model (Egger's test: *p* = 0.24; Begg's test: *p* = 0.2), recessive model (Egger's test: *p* = 0.9; Begg's test: *p* = 0.79), and dominant model (Egger's test: *p* = 0.27; Begg's test: *p* = 0.36). In addition, on inspection of funnel plot, publication bias was not obvious. Funnel plot for detection of publication bias in allelic, recessive model, and dominant model is demonstrated in Figures 5, 6, 7 respectively.

### 3.4 Sensitivity test, subgroup analysis, and meta‐regression

**Figure 8:**
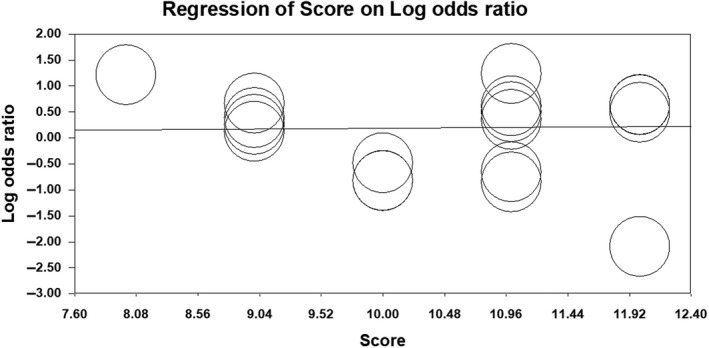
Meta‐regression plot for allelic model

**Figure 9:**
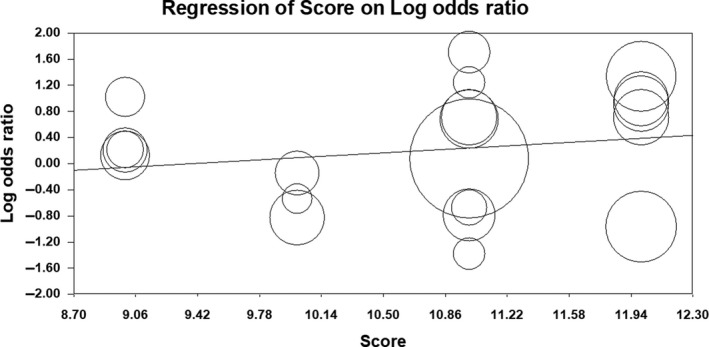
Meta‐regression plot for recessive model

**Figure 10:**
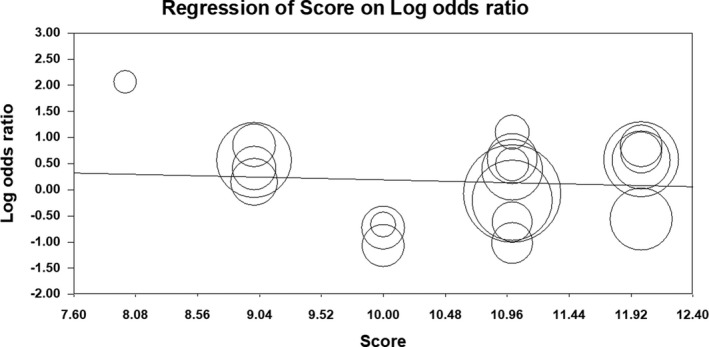
Meta‐regression plot for dominant model

For all three genetic models, we sequentially excluded single study from the pooled analysis and recalculated the summary ORs to check whether the summary ORs were significantly changed. The recalculated ORs were similar indicating stability of analysis. We performed subgroup analysis based on ethnicity and source of recruitment of controls. In all three genetic models, subgroup analysis showed that K469E polymorphism of ICAM‐1 gene was associated with ischemic stroke in the Caucasians population. Similarly in all three models of inheritance, the subgroup analysis based on source of recruitment of controls showed that K469E polymorphism was associated with ischemic stroke in subgroup of studies that recruited controls from hospital setting. The details of subgroup analysis can be seen in Table 2. We used meta‐regression analysis to explore whether methodological quality scores of included studies play an important role in the association between K469E polymorphism and ischemic stroke risk. In all three genetic models, we found that quality scores were not significant predictors of the effect size for the association between K469E polymorphism and ischemic stroke risk (allelic: *p* = 0.66, recessive: *p* = 0.06, and dominant: *p* = 0.24). Meta‐regression plot is shown in Figures 8, 9, 10.

**Table 2:** Subgroup analysis based on ethnicity and recruitment of controls under different genetic models

Subgroups			Allelic model	Recessive model	Dominant model
Ethnicity	Caucasians	No of Studies	5	6	6
		Effect size	OR: 1.86; 95% CI: 1.09 to 3.17; *p* = 0.02	OR: 2.17; 95% CI: 1.08 to 4.36; *p* = 0.03	OR: 1.71; 95% CI: 1.0 to 2.9; *p* = 0.048
		Heterogeneity	*I* ^2^ = 88%	*I* ^2^ = 87%	*I* ^2^ = 88%
	Asians	No of Studies	14	13	14
		Effect size	OR: 0.93; 95% CI: 0.63 to 1.38; *p* = 0.72	OR: 1.1; 95% CI: 0.71 to 1.72; *p* = 0.66	OR: 1.25; 95% CI: 0.88 to 1.78; *p* = 0.21
		Heterogeneity	*I* ^2^ = 94%	*I* ^2^ = 79%	*I* ^2^ = 85%
	Blacks	No of Studies	N/A	1	1
		Effect size	N/A	OR: 0.81; 95% CI: 0.6 to 1.10; *p* = 0.18	OR: 0.81; 95% CI: 0.6 to 1.10; *p* = 0.18
		Heterogeneity	N/A	N/A	N/A
Source of controls	Hospital based	No of Studies	4	4	4
		Effect size	OR: 1.56; 95% CI: 1.21 to 2.01; *p* = 0.0007	OR: 2.08; 95% CI: 1.13 to 3.8; *p* = 0.02	OR: 1.63; 95% CI: 1.25 to 2.12; *p* = 0.0003
		Heterogeneity	*I* ^2^ = 53%	*I* ^2^ = 67%	*I* ^2^ = 10%
	Community based	No of Studies	15	16	17
		Effect size	OR: 1.01; 95% CI: 0.66 to 1.54; *p* = 0.98	OR: 1.09; 95% CI: 0.74 to 1.62; *p* = 0.66	OR: 1.27; 95% CI: 0.92 to 1.75; *p* = 0.15
		Heterogeneity	*I* ^2^ = 94%	*I* ^2^ = 80%	*I* ^2^ = 88%

## 4 DISCUSSION

Atherosclerosis is a consequence of chronic inflammation and healing response of the arterial wall to endothelial injury (Libby, 2002). Dysfunctional endothelial cells express adhesion molecules like vascular cell adhesion molecules (VCAM) and ICAM‐1 which binds to LFA‐1 of monocytes and T lymphocytes and help them migrate into intima under the influence of locally produced chemokines (Wee et al., 2009). Monocytes transform into macrophages in the intima and phagocytose lipoproteins including oxidized lipoproteins and transform into lipid‐laden foam cells. These oxidized LDL further augments macrophage activation and cytokine production starting a vicious cycle of leukocyte adhesion and cytokine production (Libby, 2002, 2012). In addition, both naive and oxidized LDL increase ICAM‐1 expression in damaged blood vessels and promote atherosclerosis (Curtis et al., 2011; Kita et al., 2001). Studies have shown that ICAM‐1 mediated neutrophil adhesion plays an important role in the pathophysiology of evolutionary stroke (Connolly et al., 1996). ICAM‐1 knockout mice show reduced leukocyte adhesion, small infarction, improved cerebral blood flow, and reduced mortality after cerebral ischemia and reperfusion (Kitagawa et al., 2003). Clinical studies have shown increased expression of ICAM‐1 in brain and increased circulating levels of soluble ICAM‐1 and cerebrospinal fluid ICAM‐1 following ischemic stroke (Bitsch, Klene, Murtada, Prange, & Rieckmann, 1998; Deng et al., 2005; Ferrarese et al., 1999; Love & Barber, 2001). In addition, soluble ICAM‐1 levels are also associated with neurological deterioration following ischemic stroke (Deng et al., 2005). All of the aforementioned studies suggest that ICAM‐1 may be involved in the pathogenesis of ischemic stroke.

The K469E polymorphism is a non‐synonymous SNP located in the fifth immunoglobulin‐like domain of ICAM‐1, which is required for protein dimerization, surface presentation, and solubilization (Miller et al., 1995; Vora et al., 1994). It results in a change in glutamate (E) to lysine (K) in the immunoglobulin‐like domain 5 of the ICAM‐1 protein, which is critical for the activity of the ICAM‐1 protein to interact with LFA‐1 (Zhao et al., 2013; Wee et al., 2009). In addition, one study showed that this polymorphism affects ICAM‐1 mRNA splicing patterns and TPA‐induced apoptosis (Iwao, Morisaki, & Morisaki, 2004). Recent studies have shown a strong correlation between the K469E polymorphism and baseline soluble ICAM‐1 levels (Paré et al., 2011; Zee et al., 2007). Therefore, the K469E polymorphism of the ICAM‐1 gene may strongly influence the expression and biological activity of ICAM‐1 and have a potentially important role in the inflammation, atherosclerosis, and pathogenesis of ischemic stroke.

In the present meta‐analysis, we investigated the association between K469E polymorphism of ICAM‐1 gene and risk of ischemic stroke. Meta‐analysis found that in allelic model, recessive model, and dominant model of inheritance, K469E polymorphism in the ICAM‐1 gene is not associated with ischemic stroke susceptibility. The results of all genetic models are consistent with previously published meta‐analyses that include only the Chinese population (Ming‐Jie Zhang et al., 2014). After conducting the subgroup analysis by ethnicity, the K469E polymorphism was found to be a risk factor for ischemic stroke in Caucasian population in all the three models of inheritance. Different genetic backgrounds in Caucasians, Asians, and Blacks might contribute to this inconsistency. Of the 20 studies included, only 6 were Caucasian studies, which may have affected the true association of Caucasian ICAM‐1 polymorphism with ischemic stroke. (Flex et al., 2004; Li et al., 2009; Mohy et al., 2015; Motawi et al., 2012; Pola et al., 2003; Volcik et al., 2010). Thus, additional studies with larger cohorts are necessary. Although not statistically significant, Blacks with the K469E polymorphism showed a reduced risk of ischemic stroke. However, only one study included the Black population and the results cannot be generalized (Volcik et al., 2010). Further large scale studies with Black subjects are needed to clarify this association. In all three models of inheritance, the subgroup analysis based on source of recruitment of controls showed that K469E polymorphism was associated with ischemic stroke in subgroup of studies that recruited controls from hospital setting. The reason for strong association seen in subgroup of studies that recruited controls from hospital setting can be that half of the studies in this subgroup are from Europe with Caucasian population. Thus ethnicity can be a confounder in this association.

In addition to the K469E polymorphism of the ICAM‐1 gene, a study has shown that the ICAM‐1 gene G1548A polymorphism is an independent risk factor for the development of ischemic stroke (Lu, Liu, Wu, Chen, & Hwang, 2013). In accordance to our study, a meta‐analysis showed that the K469E polymorphism in the ICAM‐1 gene may increase individuals' susceptibility to diabetic microvascular complications, including diabetic nephropathy and diabetic retinopathy. However, in their research, the susceptibility of Asians was far‐reaching, which contradicts our findings (Su et al., 2013). Similarly, a meta‐analysis by Shengqiang et al. showed that the ICAM‐1 gene K469E polymorphism may be a risk factor for coronary artery disease (Zou et al., 2014). Flex et al. also found that the ICAM‐1 gene K469E polymorphism was significantly and independently associated with peripheral arterial occlusive disease (PAOD) (Flex et al., 2007). Similarly, Gaetani et al. showed that the presence of the EE genotype significantly independently increased the risk of PAOD (Gaetani et al., 2002). Popović et al in their study found that the EE genotype of the K469E polymorphism of the ICAM‐1 gene was associated with a more rapid progression of carotid atherosclerosis in patients with type 2 diabetes mellitus than other genotypes (Gaspar et al., 2016).

Statistically significant heterogeneity across studies was observed in all genotype models (*I* ^2^ ranged from 82% to 93%). Significant heterogeneity was also found in all subgroups in all genotype models (*I* ^2^ ranged from 53% to 94%), except in hospital based subgroup of dominant model. There could be several reasons for these heterogeneities. First, most of these studies had small sample size. Second, different strategies were applied in various studies, including control source, population stratification, phenotype selection, genotyping methods, etc. Moreover, heterogeneity may also be due to the variations in ethnicity, age, and environmental factors.

Our study has several advantages. First, this is the largest meta‐analysis on this issue, including 20 studies and 3,137 cases and 15,382 controls. Second, we included all Caucasian, black, and Asian populations to demonstrate the association between the K469E polymorphism and ischemic stroke. Third, we conducted a comprehensive search, not limited to English literature. We searched the Chinese database and extracted 13 papers in Chinese. Fourth, in most of the included studies, control subjects did not deviate from the Hardy–Weinberg equilibrium. Fifth, we performed our analysis in allelic, recessive, and dominant model of inheritance. Finally, in our analysis, publication bias is not obvious.

There were a few limitations in our study. First, some studies included in the meta‐analysis had small sample size and may have provided inconsistent results due to low statistical power. Second, stroke risk varies as per specific subtypes of stroke; however, none of the studies included in the meta‐analysis presented the data of subtype of stroke; therefore, meta‐analysis based on stroke subtype could not be performed. Third, significant heterogeneity was evident in our meta‐analysis. Lastly, multiple factors can influence the development of ischemic stroke, especially the gene‐gene interaction and gene‐environment interaction, which were not analyzed due to lack of sufficient data.

Despite these limitations, our meta‐analysis indicated that Caucasians with the ICAM‐1 gene K469E polymorphism are susceptible to ischemic stroke. In order to explore clear conclusions, further elaborate large‐scale epidemiological studies, including all ethnic groups from different geographic locations are required to validate these findings.

## 5 CONCLUSION

Evidence suggests that K469E polymorphism of ICAM‐1 is a risk factor for ischemic stroke susceptibility among Caucasians. There is hope that K469E polymorphism may serve as a marker of risk for multigenic complex disease ischemic stroke and might help in early identification of at risk individuals and form preventive strategies. Considering the importance of ICAM‐1 pathogenesis of ischemic stroke, further larger studies with gene–environment interactions in diverse ethnicity should be conducted to clarify the association between K469E polymorphism and ischemic stroke susceptibility. Furthermore, the underlying molecular causal pathways that confer susceptibility to ischemic stroke are warranted to be established to identify target for prevention and treatment of ischemic stroke.

## CONFLICT OF INTEREST

The authors declare no conflicts of interest.

## AUTHORS’ CONTRIBUTIONS

GN and YK designed the study. GN, YK and JY carried out literature search, review and selection. GN and YK carried out the statistical analysis and drafted the manuscript. All authors were involved in revising the manuscript critically for important intellectual content. All authors read and approve the final manuscript.

## ETHICAL APPROVAL

This article does not contain any studies with human participants or animals performed by any of the authors.

## INFORMED CONSENT

For this type of study formal consent is not required.
